# Dr. James A. Egan

**Published:** 1913-05

**Authors:** 


Dr. James A. Egan, N. W. University, 1893, for sixteen years Secretary of the Illinois State Board of Health and well known for his good work along sanitary and educational lines, died in Springfield, Ill., March 30, aged 53, of nephritis.

